# Role of Coronary Revascularization in Patients with Ischemic Heart Disease and Heart Failure with Reduced Ejection Fraction

**DOI:** 10.3390/jcm15051941

**Published:** 2026-03-04

**Authors:** Mikel Lacalle, Pablo Bazal, Jara García Ugalderbere, Octavio Jiménez Melo, Aritza Conty, Raúl Ramallal, Guillermo Sánchez-Elvira, Valeriano Ruiz-Quevedo

**Affiliations:** 1 Faculty of Health Sciences, Medicine, Public University of Navarre, 31009 Pamplona, Spain; 2 Interventional Cardiology Unit, Department of Cardiology, University Hospital of Navarra, 31008 Pamplona, Spain

**Keywords:** heart failure, ischemic heart disease, reduced ejection fraction, percutaneous coronary intervention, coronary revascularization, coronary artery bypass grafting

## Abstract

Heart failure with reduced ejection fraction (HFrEF) is a major contributor to cardiovascular morbidity and mortality, with ischemic heart disease as the leading etiology. Despite advances in optimal medical treatment (OMT), the additional benefit of coronary revascularization in this population remains uncertain. While some studies describe a potential benefit in revascularization—particularly with coronary artery bypass grafting (CABG)—this benefit has not been consistently observed with percutaneous coronary intervention (PCI). Moreover, in this context, the role of myocardial hibernation has been called into question. Additionally, recent advances in the medical management of heart failure complicate the current interpretation of previous studies and underscore the need for new clinical trials. This review synthesizes the current evidence on the potential benefits of coronary revascularization in patients with heart failure with reduced ejection fraction (HFrEF).

## 1. Introduction

Heart failure (HF) is defined as a clinical syndrome characterized by signs and/or symptoms caused by structural and/or functional cardiac abnormalities, corroborated by elevated natriuretic peptide levels and/or objective evidence of pulmonary or systemic congestion. According to left ventricular ejection fraction (LVEF), HF is classified into three categories: HF with reduced ejection fraction (HFrEF), mildly reduced ejection fraction (HFmrEF), or preserved ejection fraction (HFpEF), corresponding to LVEF ranges of ≤40%, 41–49%, and ≥50%, respectively [1]. The estimated prevalence of HF in adults is 1–2%, increasing progressively with age and exceeding 10% among individuals older than 70 years [2].

Ischemic heart disease is the leading underlying cause of HFrEF, accounting for approximately two thirds of all HF cases [3]. Compared with non-ischemic etiologies, ischemic HF is associated with a higher risk of all-cause mortality, mainly driven by non-sudden cardiac death [4,5]. However, distinguishing ischemic from non-ischemic HFrEF can be challenging, and concomitant coronary artery disease (CAD) is not infrequently present without being the primary cause of ventricular dysfunction. To standardize definitions, ischemic cardiomyopathy is defined as LV dysfunction with significant CAD and at least one of the following: prior revascularization/AMI, >75% stenosis of the LMCA or LAD, or multi-vessel disease (≥2 vessels with >75% stenosis) [6].

Left ventricular dysfunction in ischemic heart disease may result from myocyte loss or prior infarction, with residual scar or fibrosis. Ischemic scar typically follows the distribution of the affected coronary artery and progresses centrifugally from the endocardium toward the epicardium, becoming transmural in the case of complete infarction [7,8]. In this context, late gadolinium enhancement in cardiac magnetic resonance (LGE-CMR) is highly useful for delineating ischemic scar after infarction and identifying viable myocardium, defined as fibrosis or late enhancement involving <50% of myocardial wall thickness [9].

However, a non-negligible proportion of patients with significant CAD and HFrEF show no clinical or imaging evidence of prior transmural infarction. In these patients, ventricular dysfunction is thought to be related to dysfunctional or hypocontractile myocardial segments following the distribution of atherosclerotic coronary arteries, but without scar or fibrosis and with preserved, albeit potentially reduced, metabolic activity [10]. This so-called “hibernating” myocardium would remain viable and downregulate contractility to match reduced oxygen supply in response to chronic ischemia [11,12]. Under this premise, subsequent observations suggested that contractile function in these segments might be reversible or improved through coronary revascularization [13].

## 2. Coronary Revascularization in Ischemic Heart Disease

Two main revascularization strategies are available: coronary artery bypass grafting (CABG) and percutaneous coronary intervention (PCI). Although both approaches increase coronary blood flow and prevent myocardial ischemia, outcomes may differ depending on anatomical complexity and the patient’s clinical context [14].

The SYNTAX study demonstrated that patients with higher anatomical complexity (SYNTAX score ≥ 33) derive greater benefit from surgical revascularization, whereas PCI may be a reasonable alternative in less complex anatomies [15]. Nevertheless, CABG has been associated with a higher risk of atrial fibrillation and stroke [15,16,17,18,19,20], as well as increased early postoperative complications, including major bleeding, arrhythmias, acute kidney injury, and rehospitalization within the first 30 days [21]. Conversely, PCI is associated with a higher likelihood of repeat revascularization and myocardial infarction during follow-up, particularly in patients with extensive CAD [17,18,22,23]. Consequently, the choice between strategies should be individualized, taking into account not only coronary anatomy but also the patient’s clinical profile and myocardial viability.

Coronary revascularization has demonstrated benefit in obstructive CAD for symptom relief and prevention of spontaneous myocardial infarction and other major adverse cardiovascular events (MACE), with some authors suggesting a potential long-term survival benefit [24,25].

According to the classical concept of myocardial hibernation, combining myocardial revascularization with optimal medical therapy (OMT) could improve left ventricular systolic function and prognosis in patients with HFrEF by restoring adequate perfusion to dysfunctional but viable myocardial segments and preventing new ischemic events. However, recent data have generated controversy regarding the routine and early revascularization of patients with HFrEF, the role of PCI versus CABG in complex coronary disease, and the value of ischemia testing in guiding revascularization decisions [14]. Paradoxically, the potential benefit of myocardial revascularization has not been confirmed in studies assessing viability-guided revascularization using LGE-CMR, dobutamine stress echocardiography (DSE), or SPECT, thereby challenging the prognostic role of myocardial hibernation [10].

Importantly, perioperative risk in HFrEF patients is substantial due to severe left ventricular dysfunction, anatomical complexity, and reduced coronary flow reserve [11]. Therefore, the benefit of coronary revascularization must be consistently demonstrated and sufficiently large to offset the significant risk of clinical and hemodynamic deterioration associated with revascularization procedures and subsequent recovery.

In this regard, in anticipation of potential hemodynamic deterioration, the appropriateness of left ventricular assist device (LVAD) implantation must always be considered to ensure safe coronary revascularization [2,26]. In candidates for revascularization with severely depressed LV function and extensive CAD, CABG or PCI may be combined with temporary mechanical support (e.g., short-term LVAD or ECMO) in those with high operative risk, whereas durable LVAD is generally reserved for patients in whom complete and effective revascularization is not feasible or has failed to restore adequate ventricular function [27].

In recent years, several studies have compared revascularization strategies with each other or with conservative management based on OMT. Among them, the most relevant to date are the STICH and REVIVED-BCIS2 trials. However, the predominance of observational data and the scarcity of randomized controlled trials have yielded heterogeneous and sometimes contradictory results.

Overall, published evidence is limited, heterogeneous in design, and not always concordant, complicating interpretation and clinical application. Nonetheless, the systematic and detailed analysis of recent evidence presented in this review allows certain conclusions to be drawn regarding the relative efficacy of these therapeutic strategies and their clinical implications.

The aim of the present article is to review the available evidence regarding the potential benefits of coronary revascularization in patients with heart failure with reduced ejection fraction (HFrEF). To this end, a narrative review was conducted, based on a structured literature search performed in the PubMed and Cochrane databases. We included randomized controlled trials, meta-analyses, and major observational registries published between 2010 and 2025 that compared revascularization strategies with each other or with OMT in patients with heart failure with reduced ejection fraction and stable CAD. Studies involving patients presenting with cardiogenic shock or undergoing revascularization in the setting of acute coronary syndromes were excluded.

The review is structured according to different clinically relevant outcomes. First, the impact of revascularization on mortality will be addressed; subsequently, the evidence related to symptomatic improvement will be analyzed; finally, the available data on potential improvement in left ventricular function and its relationship with the presence of myocardial viability will be reviewed. In light of the available evidence and current clinical practice guideline recommendations, we have included, at the end of the manuscript, a flowchart proposing a structured diagnostic and therapeutic management algorithm.

## 3. Survival Benefit of Coronary Revascularization in Ischemic Cardiomyopathy with HFrEF

The analysis of mortality shows heterogeneous results depending on the strategy compared (Table 1). The randomized clinical HEART trial included 138 patients with HFrEF and evidence of viable myocardium, with 69 assigned to conservative management and 69 to an invasive strategy with intent to revascularize. Of the latter group, 45 patients were ultimately revascularized (15 by PCI and 30 by CABG). After a median follow-up of 59 months (IQR: 48–68), 36% of patients receiving OMT died, compared to 27% of those revascularized by PCI and 30% of those who underwent CABG. At 6 months, there were no significant differences in quality of life between treatment groups, as assessed by the EuroQoL EQ-5D questionnaire (mean difference: −0.023 points [95% CI: −0.144 to 0.097]) and the Minnesota Living with Heart Failure (MLWHF) questionnaire (mean difference: −3.9 points [95% CI: −11.4 to 3.5]). The results were inconclusive due to early trial termination, with a final population far smaller than initially planned (138 vs. 800 participants), and a potential benefit of revascularization could not be excluded [28].

Indeed, several meta-analyses jointly evaluating both revascularization strategies concluded that revascularization significantly reduces mortality. Nevertheless, based on these studies, it cannot be determined whether the observed benefit is independent of the chosen revascularization strategy (PCI or CABG) [29,34].

PCI had been associated in previous studies and in a recent meta-analysis with a potential reduction in mortality. However, these findings were not confirmed in more recent studies [30]. Among them, the largest randomized clinical trial published in this setting is the REVIVED-BCIS2 trial.

The REVIVED-BCIS2 trial was a prospective, multicenter, randomized, open-label trial designed to evaluate whether PCI plus optimal medical therapy (OMT) improves event-free survival in patients with severe ischemic cardiomyopathy over OMT alone. The primary endpoint was a composite of all-cause mortality or hospitalization for any cause.

The design specifically targeted the population most likely to benefit from revascularization according to the myocardial hibernation hypothesis. Eligibility required left ventricular systolic dysfunction (LVEF ≤ 35%), extensive CAD (defined as a British Cardiovascular Intervention Society jeopardy score ≥ 6), and demonstrable myocardial viability in at least four dysfunctional myocardial segments amenable to PCI. In this study, myocardial viability could be assessed using any imaging modality, including cardiac magnetic resonance (CMR), dobutamine stress echocardiography, positron emission tomography (PET), or single-photon emission computed tomography (SPECT). Although guidance was provided to support segmental viability assessment, the final determination of viability was based on local protocols and interpreted by site investigators.

For an estimated sample of 700 participants, 300 primary events were initially targeted to provide 85% power to detect a hazard ratio (HR) of 0.70. Several measures were implemented to safeguard internal validity, LVEF adjudication of the events

Patients were randomly assigned to PCI (347 patients) or OMT (353 patients). After a median follow-up of 41 months (IQR: 28–60), there were no significant differences between groups in the primary end point (HR 0.99 [95% CI 0.78–1.27]), or in all-cause mortality alone (HR 0.98 [95% CI 0.75–1.27]) [31]. Although fewer events than anticipated occurred (236 vs. 300 expected), the event rates were almost identical in both groups, suggesting that the neutral result likely reflects a true absence of treatment effect rather than an insufficient statistical power (Type II error) [31].

Regarding CABG, one of the most important studies to date was the STICH trial and its extension, STICHES.

The STICH trial was a multicenter randomized clinical trial comparing CABG (610 patients) with OMT (602 patients) in patients with CAD (of whom <3% had left main disease and 60% had three-vessel disease, defined as ≥50% stenosis) and left ventricular dysfunction (median LVEF 28% [IQR: 21–34] in the OMT group and 27% [IQR: 22–33] in the CABG group). After a median follow-up of 56 months (IQR: 48–68), CABG did not show a significant benefit in all-cause mortality (HR 0.86 [95% CI 0.72–1.04]) but did demonstrate a significant reduction in cardiovascular mortality (HR 0.81 [95% CI 0.66–1.00]) and in the composite of all-cause mortality or cardiovascular hospitalization (HR 0.74 [95% CI 0.64–0.85]). In subgroup analyses, patients with LVEF ≤ 27% showed significantly lower all-cause mortality in the CABG group (HR 0.77 [95% CI 0.60–0.98]), as did patients with three-vessel disease (HR 0.79 [95% CI 0.62–0.99]) [32].

Subsequently, Bonow et al. performed a subanalysis of this trial to assess whether the identification of myocardial viability by imaging influenced survival and response to CABG in patients with reduced LVEF and CAD. With a median follow-up of 5.1 years, the presence of prespecified viable myocardium was not associated with lower mortality after adjustment for baseline prognostic variables (HR 0.64 [95% CI 0.48–0.86]; *p* = 0.003 before adjustment; *p* = 0.21 after adjustment) [35].

The STICHES study extended the follow-up of STICH to a median of 9.8 years (maximum 13.4 years) and concluded that, in contrast to the initial study, CABG significantly reduced all-cause mortality (HR 0.84 [95% CI 0.73–0.97]), cardiovascular mortality (HR 0.79 [95% CI 0.66–0.93]), and the composite of mortality or cardiovascular hospitalization (HR 0.72 [95% CI 0.64–0.82]) in patients with ischemic cardiomyopathy and severe ventricular dysfunction. This difference remained significant for other outcomes, including hospitalization for any cause, hospitalization for HF, repeat revascularization, non-fatal myocardial infarction, or non-fatal stroke. Furthermore, given the degree of treatment crossover (55 patients assigned to CABG and 66 assigned to OMT crossed over), the surgical benefit may have been underestimated in the intention-to-treat analysis. In per-protocol analyses, the relative risk reduction associated with CABG was estimated to be as high as 25% compared with OMT [36]. These results may suggest that the survival benefit observed in previously mentioned meta-analyses evaluating revascularization as a whole may be largely driven by studies in which patients were treated with CABG.

In conclusion, the initial analysis of the STICH trial did not show significant differences in the primary endpoint between study groups (CABG vs. OMT) and, notably, cardiovascular mortality was even higher in the CABG group compared with the OMT group during early follow-up, likely reflecting perioperative risk. However, the survival curves progressively separated over long-term follow-up, with a clinically meaningful benefit of surgery becoming evident beyond approximately 24 months. Although the benefit was observed after a prolonged follow-up period, the hypothesis as to whether survival may or may not be directly attributable to the time elapsed since the intervention has not been specifically investigated, and any conclusions in this regard should therefore be interpreted with caution.

Importantly, this benefit was not limited to overall cardiovascular mortality but was also observed across its principal components. Sudden cardiac death occurred in 74 patients in the CABG group (12.1%) versus 99 patients in the OMT group (16.4%) (HR 0.73, 95% CI 0.54–0.99; *p* = 0.041) and death due to progressive pump failure occurred in 33 patients undergoing CABG (5.4%) compared with 49 patients receiving medical therapy alone (8.1%) (HR 0.64, 95% CI 0.41–1.00; *p* = 0.05). The observed benefit of CABG may be explained by a combined mechanism, including reduction of ischemic and arrhythmic substrate, prevention of recurrent ischemic events, and mitigation of progressive ventricular dysfunction.

Consistent with the findings of the STICH and REVIVED-BCIS2 trials, several meta-analyses and registries comparing both revascularization strategies have reported a mortality reduction associated with CABG compared with PCI, as well as reductions in other events such as myocardial infarction or repeat revascularization. However, the results of these meta-analyses are largely influenced by the STICH/STICHES trials, given the limited number of randomized clinical trials in this setting [17,18,22,30,33].

Among the most recent registries, the study published by Bloom et al. deserves mention, as it represents one of the largest worldwide and highlights the long-term benefits associated with CABG in patients with ischemic cardiomyopathy and HFrEF, albeit with an increased risk of periprocedural stroke and longer hospital stay [16].

The FAME 3 trial is one of the few randomized clinical trials comparing both revascularization strategies. Published in 2022, it was a non-inferiority randomized trial comparing FFR-guided PCI (757 patients) with CABG (743 patients) in patients with three-vessel disease, of whom 17.8% had LVEF ≤ 50% (18.2% in the PCI group and 17.6% in the CABG group) [21]. After one year of follow-up, the primary endpoint (a composite of all-cause mortality, myocardial infarction, stroke, or repeat revascularization) was significantly higher in the FFR-guided PCI group (HR 1.5 [95% CI 1.1–2.2]), failing to meet the non-inferiority margin (*p* = 0.35 for non-inferiority), regardless of LVEF [21]. This study is relevant because it incorporated routine use of FFR to guide PCI and employed contemporary drug-eluting stents, representing recommended revascularization practices associated with improved short- and long-term outcomes compared with angiography-guided PCI or OMT alone [37,38]. Among its limitations, the reported follow-up was limited to one year, whereas the potential long-term benefit of CABG has been demonstrated in studies such as STICHES (33). In addition, FAME 3 excluded patients with severe left ventricular dysfunction (LVEF < 30%) [21].

The survival benefit associated with CABG is thought to be partly related to a “collateralization effect,” whereby bypass grafts provide protection against future myocardial injury beyond diseased epicardial territories in the event of plaque rupture or vessel occlusion, events that more commonly occur in proximal and mid segments of epicardial vessels [39,40]. Furthermore, grafts—particularly arterial grafts—may remain patent for longer periods compared with PCI-treated coronary lesions [41].

In contrast, PCI treats only the focal atherosclerotic area where the stent is implanted, limiting its preventive effect or even creating a substrate for future events. Indeed, even if the treated segment remains permanently patent, myocardial ischemia may still occur in other segments of the same coronary artery.

When drawing conclusions regarding the optimal management of patients with ischemic cardiomyopathy and HFrEF, an important question is whether the main studies discussed are truly comparable. The two most relevant trials, REVIVED-BCIS2 and STICH, differ in several key aspects (Table 2). In REVIVED-BCIS2, patients were on average 10 years older than those in STICH, had a higher prevalence of chronic kidney disease (16% vs. 8%), and had experienced fewer prior myocardial infarctions (50% vs. 78%). In addition, fewer patients had three-vessel disease (38% vs. 60%), and a higher proportion were asymptomatic for angina at baseline (66% vs. 36%) [42]. By enrolling older patients with more comorbidities than the STICH trial, REVIVED provides evidence for a demographic that more closely reflects the “real-world” heart failure population. However, external validation could be somehow compromised, taking into account that the enrolled population represents a selected subgroup (severe LV dysfunction, extensive coronary artery disease, mostly asymptomatic, demonstrable viability, and stable clinical status under contemporary heart failure therapy). Therefore, extrapolation to patients with moderate/severe symptoms, acute coronary syndromes, advanced frailty, incomplete viability assessment, or different revascularization strategies should be undertaken with caution.

Another point to have in consideration was the rate of complete revascularization. In REVIVED, complete anatomical revascularization was 71%, according to the authors, which could be slightly higher from a functional standpoint. Therefore, a non-negligible proportion of patients underwent incomplete revascularization, either due to anatomical complexity, diffuse disease, or clinical decision-making. This may be relevant, as it could have contributed to the neutral result of the study, and has been described as a potential limitation when comparing outcomes with CABG studies [42,43].

Furthermore, as mentioned before, the heterogeneity in imaging modalities employed in viability assessment—each with different diagnostic thresholds, sensitivities, and center-specific criteria for defining viable myocardium—together with potential interobserver variability, may have introduced misclassification. Such variability could have attenuated the observed treatment effect and contributed to a dilution of the potential benefit of PCI.

Under these premises, the lesser benefit of PCI may be explained by a lower amount of myocardium at risk, reduced functional reserve, variability in myocardial viability assessment, incomplete revascularization, and higher all-cause mortality driven by key predictors such as age and chronic kidney disease. Ultimately, a higher burden of comorbidities—and consequently a greater impact of competing risks—may have contributed to an underestimation of the potential benefit of PCI [42].

Two further key differences between both trials should be highlighted. First, patients in REVIVED-BCIS2 were followed for a shorter period than those in STICH, potentially missing a late survival benefit of PCI similar to that observed with extended follow-up in STICHES, and may underestimate the potential benefit of percutaneous revascularization. Moreover, given the lower extent of CAD, it cannot be excluded that some REVIVED-BCIS2 patients had left ventricular dysfunction not clearly attributable to ischemic etiology, which may also have influenced the lower observed efficacy of revascularization.

Second, patients in REVIVED-BCIS2 received more contemporary HF medical therapy (90% received renin–angiotensin–aldosterone system inhibitors, 90% beta-blockers, and 48% mineralocorticoid receptor antagonists). Agents such as ARNIs or SGLT2 inhibitors were not available during STICHES or the early phase of REVIVED-BCIS2. In addition, patients in REVIVED-BCIS2 were more frequently treated with ICD/CRT (21%/53% vs. 2%/19%). This is a key consideration when interpreting the results of both studies, as mortality and HF hospitalization rates were significantly lower in patients treated with OMT alone in REVIVED-BCIS2 compared with any of the STICHES groups [42].

Based on the above, although the most relevant studies to date have not demonstrated that PCI improves survival, comparisons between revascularization strategies (PCI vs. CABG) should be interpreted with caution. Moreover, to date, no randomized clinical trials have specifically compared both strategies head-to-head, and observational results are not exempt from residual confounding, thus comparisons across studies remain speculative and should primarily be considered hypothesis-generating [42]. 

Considering all the limitations in the available evidence, and despite the demonstrated lack of overall benefit, there remains a clear consensus within the contemporary cardiovascular scientific community regarding the fundamental role and potential benefit of percutaneous coronary intervention (PCI) in selected patients, particularly in those with non-complex coronary anatomy in whom surgery may be considered disproportionate (e.g., one- or two-vessel CAD), or in those with prohibitive surgical risk.

## 4. Effect of Revascularization on Ventricular Function: Myocardial Hibernation and Viability Assessment

One of the potential benefits of revascularization, in relation to the myocardial hibernation hypothesis, would be improvement in ventricular function. Although viability is often identified with hibernation, it should be noted that these are not interchangeable or synonymous terms. In the context of HFrEF, contractile alterations may occur in viable myocardium secondary to mechanisms other than hibernation (e.g., stunning or negative remodeling), which may therefore not improve with myocardial revascularization [10].

Furthermore, current imaging tests, in addition to the limitations previously mentioned in detecting myocardial viability due to heterogeneity in defining criteria, face additional challenges in identifying viable tissue as specifically hibernated.

Cardiac magnetic resonance (CMR) is widely used for the assessment of myocardial viability, with late gadolinium enhancement (LGE) allowing accurate quantification of scar burden. Viability is commonly defined using a threshold of <50% infarct transmurality. However, this parameter may underestimate hibernating myocardium, or even misdiagnosed CAD etiology, in the absence of overt scar and has shown limitations in predicting contractile recovery within non-scarred segments [9]. Contractile reserve is better assessed by means of dobutamine stress imaging. The predictive accuracy for functional recovery improves when LGE-CMR is combined with dobutamine stress CMR, particularly in myocardial segments with intermediate degrees of transmurality (25–75%) [44].

In a prespecified secondary analysis of the REVIVED-BCIS2 trial (including 87% of patients from the original trial), the investigators sought to determine whether a significant correlation existed between the extent of viable myocardium, assessed by LGE-CMR or dobutamine stress echocardiography. The median increase in LVEF was 4.7% (IQR: −2.2% to 12.5%) at 6 months; however, this improvement was not determined by the extent of viable myocardium and did not differ significantly between the PCI group and the group receiving OMT alone [45].

Accordingly, the results of this study did not support the use of myocardial viability assessment to guide revascularization in patients with HFrEF [45]. The authors suggested that current viability imaging techniques may not specifically detect myocardial hibernation or even that the hibernation paradigm itself may require revision, as although ischemia may trigger the hibernation process, revascularization alone may not be sufficient to effectively reverse it [46,47].

Nevertheless, this secondary analysis of REVIVED-BCIS2 did show that a greater extent of non-viable myocardium was associated with lower event-free survival, driven by a higher incidence of fatal ventricular arrhythmias. This underscores, irrespective of the concerns raised regarding the sensitivity of hibernating myocardium diagnosis, the ongoing importance of assessing the presence and extent of myocardial scar as an integral component of the diagnostic workup and risk stratification in these patients [45].

The PARR-2 (PET and Recovery Following Revascularization-2) trial was a prospective, multicenter, randomized controlled study designed to evaluate whether a management strategy guided by myocardial viability assessment using 18F-fluorodeoxyglucose positron emission tomography (FDG-PET) improves clinical outcomes in patients with ischemic left ventricular dysfunction (left ventricular ejection fraction ≤ 35%) and known or suspected CAD considered potential candidates for coronary revascularization. The primary endpoint was a composite of all-cause mortality or hospitalization for cardiac causes during follow-up, which extended to approximately one year. In the intention-to-treat analysis viability-guided revascularization using FDG-PET did not demonstrate a benefit compared with standard management. A reduction in cardiovascular events at 1 and 5 years was observed only in a subanalysis of centers with greater imaging expertise and in patients who closely adhered to management recommendations [48,49]. For this reason, the authors suggested that PET-based viability assessment might be useful when these conditions are likely to be met [49].

A recent publication suggests that the extent of viable myocardium, with or without contractile dysfunction, may help identify patients who derive greater benefit from revascularization. However, the authors advocate for a broader concept of viability assessment, emphasizing multidisciplinary management and optimization of OMT, for which robust evidence exists [50].

On the other hand, regardless myocardial contractility or ventricular function improvement, revascularization may enhance patient prognosis, as hibernated tissue has been associated with arrhythmogenic substrates in HFrEF predisposing patients to sudden arrhythmic death. Notably, regions with small volumes of residual viable tissue—insufficient to result in measurable functional improvement—may still represent an arrhythmogenic risk that revascularization could mitigate even when pump function recovery is unlikely [44].

In this context, the use of myocardial viability testing in patients with ischemic left ventricular dysfunction remains controversial, as randomized clinical trials have not demonstrated clear benefits of viability-guided revascularization, underscoring the current limitations in optimal patient selection. Nevertheless, multimodality imaging to assess myocardial viability and ischemia remains fundamental in contemporary clinical practice. Beyond its debated role in directly guiding revascularization, it is essential for clarifying the etiology of cardiomyopathy, risk stratification, arrhythmic risk assessment, and prognostic evaluation, thereby supporting comprehensive clinical decision-making. Moreover, although evidence supporting viability-guided revascularization is limited, the expectation of functional recovery after revascularizing non-viable myocardium is biologically implausible. In complex patients with extensive coronary artery disease, imaging findings may therefore help prioritize target territories for revascularization and refine individualized therapeutic strategies.

## 5. Effect of Revascularization on Other Clinical Outcomes

### 5.1. Symptomatic Improvement

Another rationale for considering revascularization in patients with HFrEF is symptomatic improvement and, consequently, enhanced quality of life, either through improved ventricular function or reduced ischemia during daily activities. This benefit was demonstrated in earlier trials such as COURAGE and ISCHEMIA; however, these studies systematically excluded patients with HFrEF (mean LVEF 61% and 60%, respectively) [51,52].

In the REVIVED-BCIS2 trial, a significant improvement in quality of life was observed at 6 months, but this benefit was no longer present after 24 months of follow-up. A key limitation of the study was that 66% of patients were angina-free and 77% were in NYHA class I or II prior to revascularization [31].

Therefore, these results cannot be extrapolated to patients whose angina significantly impairs quality of life or to those presenting with acute coronary syndromes. PCI should not be recommended solely to achieve short-term symptomatic improvement, as although a significant benefit was observed during the first six months after revascularization, this effect was not sustained at 1 and 2 years of follow-up. A possible explanation for the loss of long-term benefit is that PCI initially improves ischemia and its clinical manifestations, leading to a subjective perception of improved well-being. Over time, however, progression of atherosclerotic disease—either in the treated vessel or in remote territories—may attenuate this initial benefit, resulting in convergence of health status with patients treated with OMT alone [31,53].

Similarly, the HEART study failed to demonstrate a significant improvement in quality of life in revascularized patients compared with those treated with OMT alone. However, most enrolled patients had minimal symptoms or had modified their lifestyle to avoid symptom onset, making it more difficult to detect improvements during follow-up [28].

With regard to specific subpopulations of particular interest, the greatest symptomatic benefit was observed among patients with more severe baseline symptoms, particularly those with severe or daily angina. The symptomatic benefit appears to be independent of age. In the STICH trial, modest improvements in quality of life, as assessed by the Minnesota Living with Heart Failure Questionnaire (MLHFQ), were observed with CABG in patients ≥ 70 years of age, with greater angina relief compared with medical therapy alone. Similarly, subgroup analyses from the ISCHEMIA and COURAGE trial in elderly patients (≥75 years and ≥65 years respectively) demonstrated improvement in angina frequency [51,52].

Regarding comorbidity burden, in STICH, patients with ≥2 comorbidities (e.g., diabetes mellitus or chronic kidney disease) derived a symptomatic benefit from CABG comparable to that observed in the overall study population, with significant improvement in KCCQ scores at 12 months and no significant interaction effects. In contrast, in ISCHEMIA, multimorbid patients experienced angina relief following revascularization but were at higher risk of adverse clinical events [32,52]. In conclusion, across major contemporary trials evaluating revascularization strategies, symptomatic benefit in elderly patients and those with multiple comorbidities has generally been preserved, although its magnitude and durability vary according to the intervention and clinical context, with a greater impact in those with severe symptoms.

### 5.2. Heart Failure Hospitalizations and Other Major Adverse Cardiovascular Events

Overall, studies including PCI as a treatment strategy did not demonstrate significant differences compared with conservative management with OMT [29,31,34,54]. In contrast, when CABG was the selected revascularization strategy, as in the STICH trial and its extension STICHES, a reduction in cardiovascular hospitalizations—particularly for heart failure—was observed. This benefit emerged early and increased over time during the 10-year follow-up [32,36,55].

## 6. Current Recommendations and Future Perspectives

Current European Society of Cardiology (ESC) guidelines highlight the complexity of revascularization decisions in patients with chronic coronary syndrome and HFrEF, recommending individualized evaluation by a multidisciplinary Heart Team. Based on available evidence, CABG is more strongly recommended to improve long-term prognosis in patients with multivessel disease who are suitable surgical candidates (Class I-C), whereas PCI may be considered in patients at high surgical risk or deemed inoperable (Class IIb-B). Additionally, coronary revascularization is recommended for symptom relief in patients with persistent angina or angina equivalents despite OMT (Class I-A).

**Figure 1:**
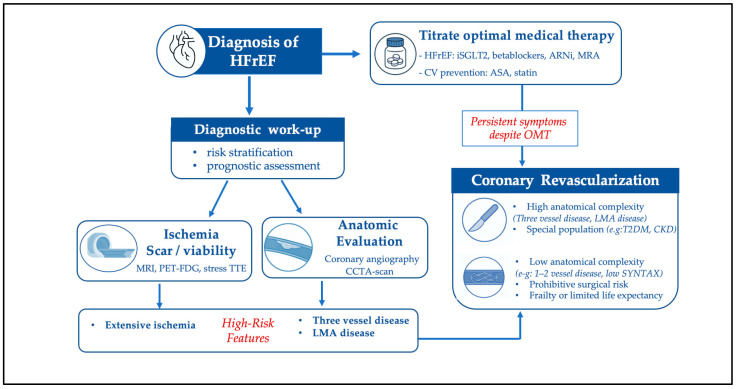
Proposed flow chart for the clinical management of patients with HFrEF. HFrEF: heart failure with reduced ejection fraction; SGLT2i: sodium–glucose cotransporter 2 inhibitors; ARNI: angiotensin receptor–neprilysin inhibitor; MRA: mineralocorticoid receptor antagonist; ASA: acetylsalicylic acid; OMT: optimal medical therapy; MRI: magnetic resonance imaging; PET: positron emission tomography; FDG: fluorodeoxyglucose; TTE: transthoracic echocardiography; CCTA: coronary computed tomography angiography; LMA: left main coronary artery; T2DM: type 2 diabetes mellitus; CKD: chronic kidney disease.

Based on the available scientific evidence and clinical practice guidelines outlined above, we propose a practical approach for managing patients diagnosed with HFrEF, as summarized in Figure 1. The main indications for revascularization are similar to those in the general population with chronic coronary syndrome—either symptomatic or prognostic—driven by high-risk features identified on diagnostic or anatomical evaluation.

It is essential, within the context of the diagnostic assessment, to describe the presence and extent of myocardial scarring or the absence of myocardial viability—particularly as evaluated by late gadolinium enhancement on cardiac magnetic resonance imaging—as these findings are closely associated with the prognosis of patients with ischemic heart disease and heart failure with reduced ejection fraction [56]. In selected high-risk patients, LVAD support may also be used to stabilize hemodynamics and end-organ function before or after complex coronary revascularization, although this strategy is reserved for highly specialized centers [57].

Despite the available evidence, uncertainty remains regarding the incremental benefit of coronary revascularization over OMT alone in patients with HFrEF, even in the context of recent advances in medical therapy and PCI techniques. Ongoing randomized multicenter trials with adequate statistical power, such as STICH3-BCIS4 (NCT05427370) and STICH-SWEDEHEART (NCT05329285), which directly compare PCI and CABG in patients with multivessel disease and HFrEF, are expected to clarify optimal revascularization strategies in clinical practice [58,59].

## 7. Conclusions

Myocardial revascularization with CABG has shown a long-term prognostic benefit over conservative management with OMT in selected patients with ischemic cardiomyopathy and HFrEF, particularly those with multivessel disease, although this advantage emerges only over extended follow-up and must be weighed against the upfront surgical risk. PCI, while often associated with transient symptomatic relief and quality-of-life improvement, has not consistently demonstrated clear reductions in mortality, sustained increases in LVEF, or major adverse cardiovascular events compared with OMT, especially in the context of contemporary guideline-directed medical therapy that may attenuate the incremental benefit of any revascularization strategy.

Although contemporary evidence tends to favor surgical revascularization with coronary artery bypass grafting (CABG), the optimal therapeutic strategy should be determined by a multidisciplinary Heart Team. This decision-making process should incorporate coronary anatomy, clinical presentation, comorbidity burden, and the overall clinical context in order to individualize the choice between CABG and percutaneous coronary intervention (PCI) for each patient.

The role of myocardial viability testing to guide revascularization in patients with HFrEF remains controversial, as it has not reliably predicted functional recovery or prognostic improvement, thereby challenging the traditional paradigm of myocardial hibernation.

## Figures and Tables

**Table 1:** Main clinical trials evaluating the impact of coronary revascularization in HFrEF.

Author and Year/Study Type	Main Comparison	Population Size	Main Results
Cleland et al. (2011) [28] *Randomized Controlled Trial*	PCI/CABG vs. OMT	114 patients (69 OMT, 15 PCI, 30 CABG)	Median follow-up of 59 months: Similar total mortality and quality of life at 6 months in both groups.
Al-Sadawi et al. (2024) [29] *Meta-analysis*	PCI/CABG vs. OMT	10,110 patients (3752 PCI/CABG, 6358 OMT)	Mean follow-up of 39 months: Significant reduction in total mortality (OR 0.56 [CI 95% 0.46–0.69]) and CV mortality (OR 0.54 [CI 95% 0.40–0.74]) with revascularization. Greater reduction if viable myocardium is present. No significant differences in HF hospitalization or MI.
Wolff et al. (2017) [30] *Meta-analysis*	PCI vs. CABG; CABG vs. OMT; PCI vs. OMT	931 (PCI vs. OMT); 6896 (CABG vs. OMT); 8782 (PCI vs. CABG)	Median follow-up of 36 months: CABG significantly reduced mortality (HR 0.82 [CI 95% 0.75–0.90]), MI (HR 0.50 [CI 95% 0.36–0.68]), and repeat revascularization (HR 0.34 [CI 95% 0.24–0.47]) vs. PCI. Both PCI (HR 0.73 [CI 95% 0.62–0.85]) and CABG (HR 0.66 [CI 95% 0.61–0.72]) reduced mortality vs. OMT.
Perera et al. (2022) [31] *Randomized Controlled Trial (REVIVED-BCIS)*	PCI vs. OMT	700 patients (347 PCI, 353 OMT)	Median follow-up of 41 months: No significant differences in mortality or HF hospitalization (HR 0.99 [CI 95% 0.78–1.27]). Improved Quality of Life (KCCQ) at 6 and 12 months, but not at 24. No significant improvement in LVEF at 12 months.
Velazquez et al. (2011/2016) [32] *Randomized Controlled Trial(STICH/STICHES)*	CABG vs. OMT	1212 patients (610 CABG, 602 OMT)	Median follow-up of 56 months: Significant reduction in CV death/hospitalization (HR 0.74 [CI 95% 0.64–0.85])/Median follow-up 9.8 years a significant reduction in all-cause mortality and CV death/hospitalization (HR 0.72 [CI 95% 0.64–0.82]) in the CABG group.
Park et al. (2020) [33] *Retrospective Cohort*	PCI vs. CABG	3488 patients (2133 PCI, 1355 CABG)	Median follow-up 3.8 years: Risk of primary outcome (mortality/MI/stroke) significantly higher in PCI group for patients with LVEF < 35% (HR 2.45 [CI 95% 1.27–4.73]) and 35–45% (HR 2.23 [CI 95% 1.17–4.28]). No difference if LVEF ≥ 45%.
Fearon et al. (2022) [21] *Randomized Controlled Trial (FAME 3)*	FFR-guided PCI vs. CABG	1500 patients (757 PCI, 743 CABG)	1-year follow-up: Risk of primary outcome (death/MI/stroke/repeat revasc) significantly higher in FFR-PCI group (HR 1.5 [CI 95% 1.1–2.2]). No significant difference in the LVEF ≤ 50% subgroup (HR 1.3 [CI 95%: 0.7–2.6]).
Bloom et al. (2024) [16] *Retrospective Cohort*	PCI vs. CABG	2042 patients (591 PCI, 1451 CABG)	Median follow-up of 4 years: Significant long-term mortality reduction in the CABG group (HR 0.59 [CI 95% 0.45–0.79]). However, in patients with LVEF < 30%, mortality was higher in the CABG group (HR 1.40 [CI 95% 1.19–1.65]).
Yu et al. (2023) [17] *Meta-analysis*	PCI vs. CABG	11,032 patients (5521 PCI, 5511 CABG)	Follow-up 30 days to 15 years: No overall difference in all-cause or CV mortality. In matched studies, PCI was associated with higher mortality (RR 1.19 [CI 95% 1.10–1.28]), MI (RR 1.99 [IC 95% 1.02–3.88]), HF (RR 1.29 [CI 95% 1.17–1.43]), and repeat revascularization (RR 2.74 [CI 95% 1.93–3.90). PCI had lower stroke/TIA risk (RR 0.71 [CI 95% 0.58–0.86]).

CABG: coronary artery bypass grafting. OMT: optimal medical therapy, PCI: percutaneous coronary intervention, MACE: major adverse cardiovascular events, CV: cardiovascular. FFR: fractional flow reserve. HR: hazard ratio (HR). MI: acute myocardial infarction, OR: odds ratio, RR: relative risk, PET: positron emission tomography.

**Table 2:** Main differences between REVIVED-BCIS and STICH trials.

	REVIVED-BCIS	STICH/STICHES
Mean age	70 years	60 years
Chronic kidney disease	16%	8%
Prior myocardial infarction	50%	78%
Three-vessel disease	38%	60%
Angina-free at baseline	66%	36%
Follow-up (median)	41 months	56 months (STICH) 9.8 years (STICHES)
HF medical therapy	90% ACEi/ARB/ARNI 90% beta-blockers 48% MRA	90% ACEi/ARB 88% beta-blockers 46% MRA ARNI/SGLT2i not available
ICD/CRT use	21%/53%	2%/19%

## Data Availability

No new data were created in this study. Data sharing is not applicable to this review article.

## Abbreviations

The following abbreviations are used in this manuscript:

ACEi	Angiotensin-Converting Enzyme Inhibitor
AMI	Acute Myocardial Infarction
ARB	Angiotensin II Receptor Blocker
ARNI	Angiotensin Receptor–Neprilysin Inhibitor
CABG	Coronary Artery Bypass Grafting
CI	Confidence Interval
CMR	Cardiac Magnetic Resonance
CRT	Cardiac Resynchronization Therapy
DSE	Dobutamine Stress Echocardiography
ESC	European Society of Cardiology
FDG-PET	Fluorodeoxyglucose Positron Emission Tomography
FFR	Fractional Flow Reserve
HF	Heart Failure
HFmrEF	Heart Failure with Mildly Reduced Ejection Fraction
HFpEF	Heart Failure with Preserved Ejection Fraction
HFrEF	Heart Failure with Reduced Ejection Fraction
HR	Hazard Ratio
ICD	Implantable Cardioverter-Defibrillator
IQR	Interquartile Range
LAD	Left Anterior Descending (coronary artery)
LGE-CMR	Late Gadolinium Enhancement Cardiac Magnetic Resonance
LMCA	Left Main Coronary Artery
LVEF	Left Ventricular Ejection Fraction
MACE	Major Adverse Cardiovascular Events
MRA	Mineralocorticoid Receptor Antagonist
NYHA	New York Heart Association
OMT	Optimal Medical Therapy
PCI	Percutaneous Coronary Intervention
SGLT2i	Sodium–Glucose Cotransporter 2 Inhibitor
SPECT	Single Photon Emission Computed Tomography
